# A large-scale database of T-cell receptor beta sequences and binding associations from natural and synthetic exposure to SARS-CoV-2

**DOI:** 10.3389/fimmu.2025.1488851

**Published:** 2025-02-17

**Authors:** Sean Nolan, Marissa Vignali, Mark Klinger, Jennifer N. Dines, Ian M. Kaplan, Emily Svejnoha, Tracy Craft, Katie Boland, Mitchell W. Pesesky, Rachel M. Gittelman, Thomas M. Snyder, Christopher J. Gooley, Simona Semprini, Claudio Cerchione, Fabio Nicolini, Massimiliano Mazza, Ottavia M. Delmonte, Kerry Dobbs, Gonzalo Carreño-Tarragona, Santiago Barrio, Vittorio Sambri, Giovanni Martinelli, Jason D. Goldman, James R. Heath, Luigi D. Notarangelo, Joaquin Martinez-Lopez, Bryan Howie, Jonathan M. Carlson, Harlan S. Robins

**Affiliations:** ^1^ Adaptive Biotechnologies, Seattle, WA, United States; ^2^ Microsoft Research, Redmond, WA, United States; ^3^ Unit of Microbiology - The Great Romagna Hub Laboratory, Pievesestina ITALY and DIMES, University of Bologna, Bologna, Italy; ^4^ IRCCS Istituto Romagnolo per lo Studio dei Tumori (IRST) “Dino Amadori”, Meldola, Italy; ^5^ Immunotherapy, Cell Therapy and Biobank (ITCB), IRCCS Istituto Romagnolo per lo Studio dei Tumori (IRST) “Dino Amadori”, Meldola, Italy; ^6^ Immune Deficiency Genetics Section, Laboratory of Clinical Immunology and Microbiology, National Institute of Allergy and Infectious Diseases, National Institutes of Health, Bethesda, MD, United States; ^7^ Hematology Department, Hospital 12 de Octubre, i+12, CNIO, Complutense University, Madrid, Spain; ^8^ Swedish Medical Center, Seattle, WA, United States; ^9^ Division of Allergy and Infectious Diseases, University of Washington, Seattle, WA, United States; ^10^ Institute for Systems Biology, Seattle, WA, United States

**Keywords:** SARS-CoV-2, COVID-19, T cell, TCR repertoire, immune response, cellular immunity

## Abstract

We describe the establishment and current content of the ImmuneCODE™ database, which includes hundreds of millions of T-cell Receptor (TCR) sequences from over 1,400 subjects exposed to or infected with the SARS-CoV-2 virus, as well as over 160,000 high-confidence SARS-CoV-2-associated TCRs. This database is made freely available, and the data contained in it can be used to assist with global efforts to understand the immune response to the SARS-CoV-2 virus and develop new interventions.

## 1 Introduction

The emergence of SARS-CoV-2 in December of 2019 and the ensuing pandemic declared by the WHO at the end of January 2020 created an urgent need to understand the disease and its causative agent. Initial studies showed a strong T-cell based adaptive immune response to the virus (1–3), which is unsurprising given the ability of CD8+ and CD4+ T cells to recognize viral antigens presented by MHC class I and class II molecules. Antigen recognition by T cells leads to killing of infected cells, generation of T-cell memory against viral antigens, and the development of anti-viral antibodies (4). More detailed characterizations of the cellular immune response could aid the development of new interventions, soe applied our previously described Adaptive immunosequencing assay (5–7) and MIRA™ tool (8, 9) to deepen the understanding of the adaptive immune response to SARS-CoV-2 infection in support of COVID-19 research.

To generate these data, we partnered with Microsoft, Illumina, Labcorp/Covance, and health organizations across the world to generate the ImmuneCODE database described herein. These data are being made freely available to the scientific community so that any researcher, public health official or organization can utilize the data to accelerate ongoing global efforts to develop better diagnostics, vaccines, and therapeutics, as well as to answer important questions about the virus.

The database consists of two distinct but related datasets. (A) The immunosequencing dataset includes 1,486 deeply-sampled TCRβ repertoires from subjects who at the time of sampling either had been exposed to, were actively suffering from, or had recovered from COVID-19. These data originate from two sources (**Table 1**): ImmuneRACE (Immune Response Action to COVID-19 Events), a prospective study enrolling participants across the U.S. to decode how immune systems detect and respond to the virus, which includes self-reported demographic and clinical data; and more than a thousand de-identified geographically and ethnically diverse patient blood samples collected by institutions around the world. (B) The MIRA dataset maps TCRs to the SARS-Cov-2 virus epitopes they bind, and includes data obtained from exposed subjects and naïve controls. In total, the MIRA dataset includes more than 160,000 high-confidence SARS-CoV-2-associated TCRs. The data include varying degrees of demographic and clinical information (as allowed by each institution and corresponding IRB).

**Table 1:** List of available samples per dataset, including number of samples, institution and description of sample type and source.

Name of the dataset	Sample count	Institution	Study description
COVID-19-Adaptive	160	Adaptive Biotechnologies	ImmuneRACE and Antigen Map COVID19: immune response to COVID-19 (with Microsoft); cDNA or gDNA from T cells, B-cell depleted T cells, or PBMCs
COVID-19-Adaptive-MIRAMatched	72	Adaptive Biotechnologies	Whole blood samples from convalescent patients collected at Bloodworks Northwest (Seattle, WA); these samples were used for both MIRA and repertoire sequencing
COVID-19-BWNW	50	Bloodworks Northwest	Whole blood samples from convalescent patients collected at Bloodworks Northwest (Seattle, WA)
COVID-19-DLS	433	Discovery Life Sciences	Whole blood samples collected during routine patient care in acute and convalescent phases procured through Discovery Life Sciences (Huntsville, AL)
COVID-19-ISB	157	Institute for Systems Biology	Whole blood samples collected under the INCOVE project at Providence St. Joseph Health (Seattle, WA). Patients were enrolled during the active phase and monitored through disease
COVID-19-NIH/NIAID	357	National Institute for Allergy and Infectious Diseases (NIAID)	Whole blood samples were collected in Brescia, Monza and Pavia (Italy) during active infection, and provided to the NIAID (Bethesda, MD) for DNA extraction
COVID-19-HUniv12Oct	193	Hospital Universitario 12 de Octubre	Whole blood samples were collected at the Hospital Univesitario 12 de Octubre (Madrid, Spain) during the active or convalescent phase
COVID-19-IRST/AUSL	64	Istituto Scientifico Romagnolo per lo Studio e la Cura dei Tumori (IRST)/AUSL-Romagna	Whole blood samples were collected by IRST/AUSL (Romagna, Italy) during active infection

By associating T-cell signatures with disease and outcomes, the ImmuneCODE database may inform our understanding of the immune response to the virus and help researchers around the world accelerate their work in basic and applied immunology, thus contributing to the development of new therapeutic and preventive measures.

## 2 Materials and methods

### 2.1 ImmuneRACE experimental cohort and study approval

The ImmuneRACE study is a prospective, single group, multi-cohort, exploratory study of unselected eligible participants exposed to, infected with, or recovering from COVID-19 (NCT04494893). Participants, aged 18 to 89 years and residing in 24 different geographic areas across the United States, were consented and enrolled via a virtual study design. Cohorting was based on participant-reported clinical history following the completion of both a screening survey and study questionnaire.

Cohort 1 included participants exposed within 2 weeks of study entry to someone with a confirmed COVID-19 diagnosis, either based on positive PCR testing or clinician diagnosis. Cohort 2 participants included those clinically diagnosed by a physician or with positive laboratory confirmation of active SARS-CoV-2 infection via PCR testing; no participants were shared between Cohort 1 and Cohort 2. Cohort 3 included participants previously diagnosed with COVID-19 disease who have been deemed recovered based on two consecutive negative nasopharyngeal or oropharyngeal (NP/OP) PCR tests, clearance by a healthcare professional, or the resolution of symptoms related to their initial COVID-19 diagnosis. The ImmuneRACE study was approved by Western Institutional Review Board (WIRB reference number 1–1281891-1, Protocol ADAP-006). All participants were consented for sample collection and metadata use via electronic informed consent processes.

Both whole blood and serum and a nasopharyngeal or oropharyngeal swab were collected from participants by trained mobile phlebotomists. Blood samples were shipped frozen or at room temperature to Adaptive Biotechnologies for processing, including, but not limited to, DNA extraction, and TCRβ analysis via the Adaptive immunosequencing assay (Adaptive Biotechnologies, Seattle, WA) from DNA extracted from blood samples. No batch effects were observed between samples shipped frozen vs. at room temperature. NP/OP swabs and serum were sent to Covance/Labcorp for further testing. An electronic questionnaire was administered to collect information pertaining to the participant’s medical history, symptoms, and diagnostic tests performed for COVID-19 disease. Participants had the option to undergo additional blood draws and questionnaires over 2 months.

### 2.2 Global data collaborations

Whole blood samples were collected in K2EDTA tubes based on each institution’s protocol and supervised by their respective Institutional Review Board. Samples were stored at the institution and sent to Adaptive as frozen whole blood, isolated PBMC or DNA extracted from either sample type for TCRβ analysis via the Adaptive immunosequencing assay (see **Table 1**). Samples provided by the NIAID were collected under approval by Comitato Etico Provinciale (protocol NP-4000), by Comitato Etico, Ospedale San Gerardo Monza (protocol COVID-STORM) and by Comitato Etico Pavia Fondazione IRCCS Policlinico San Matteo, Pavia (protocol 20200037677). Whole blood samples from DLS (Discovery Life Sciences, Huntsville, AL) were collected under Protocol DLS13 for collection of remnant clinical samples. From Bloodworks Northwest (Seattle, WA), volunteer donors recovered from COVID-19 were consented and collected under the Bloodworks Research Donor Collection Protocol BT001. Samples were processed for PBMC and donor data reported by the Biological Products division of Bloodworks NW under standard operating procedures.

### 2.3 Sample analysis

A subset of the samples were processed for both T-cell receptor variable beta chain repertoire sequencing and MIRA, and another subset was processed only by one of these approaches. For each subject included in the dataset, *subject_id* (alternately coded as *Subject*) can be used to determine which assays were used to process which samples.

### 2.4 T-cell receptor variable beta chain sequencing

Immunosequencing of the CDR3 regions of human TCRβ chains was performed using the Adaptive assay as previously described (5–7). In brief, as much as 18 μg of extracted genomic DNA was amplified in a bias-controlled multiplex PCR with primers targeting all known TCRβ V and J genes on either side of the CDR3 region, followed by high-throughput sequencing. Sequences were collapsed and filtered in order to identify and quantitate the absolute abundance of each unique TCRβ CDR3 region, with additional bias correction performed computationally based on inline synthetic control molecules. In order to quantify the proportion of T cells out of total nucleated cells input for sequencing, or T-cell fraction, a panel of reference genes present in all nucleated cells was amplified simultaneously.

### 2.5 Multiplexed identification of TCR-antigen specificity

To identify antigen-specific TCRs, T cells derived post-expansion from either of the above input cell types were used for MIRA. Antigen-specific TCRs were identified as previously described (8, 9). Briefly, T cells were incubated overnight with MIRA peptide pools, and the antigen-specific subset was identified by CD137 upregulation. Following addition of peptides, cells were incubated at 37°C for ~18 hours. At the end of the incubation, replicate wells of cells were harvested from the culture and pooled and then stained with antibodies for analysis and sorting by flow cytometry. Cells were then washed and suspended in PBS containing FBS (2%), 1mM EDTA and 4,6-diamidino-2-phenylindole (DAPI) for exclusion of non-viable cells. Cells were acquired and sorted using a FACS Aria (BD Biosciences) instrument. Sorted antigen-specific (CD3+CD8+CD137+) T cells were pelleted and lysed in RLT Plus buffer for nucleic acid isolation; a small number of experiments sorted on CD4+ rather than CD8+ since the MIRA peptides in those experiments were selected for presentation by MHC class II molecules. Analysis of flow cytometry data files was performed using FlowJo (Ashland, OR).

RNA was isolated using AllPrep DNA/RNA mini and/or micro kits, according to manufacturer’s instructions (Qiagen). RNA was reverse transcribed to cDNA using Vilo kits (Life Technologies). TCRβ amplification, sequencing and clonotype determination were performed as described in the ‘T-cell receptor variable beta chain sequencing’ section above.

### 2.6 MIRA panel design

T-cell populations were exposed to pooled peptides or transgenes in a combinatoric format, similar to the approach described in (9). According to the MIRA panel design, each antigen is strategically placed in a subset of K unique pools while being omitted from the remaining pools (total pools = N). This design allows for antigens to be placed into a unique combination of N choose K “addresses,” where each address is a unique set of K pools; this allows for parallel testing of more antigens as the number of replicate pools (N) increases. In order to estimate an empirical false discovery rate and gauge assay quality, we left >40% of the unique addresses empty to assess the rate at which clones are spuriously sorted and detected in K pools with no query antigen present (hereafter referred to as invalid TCR associations).

### 2.7 Matching clonotypes to antigens

T cells were aliquoted into 11 pools, and activated T cells were sorted using T-cell markers after overnight stimulation, as described previously (9). These putative antigen responding cells were set aside to characterize the T-cell clonotypes present in each sorted pool using the Adaptive immunosequencing assay. After immunosequencing, we examined the behavior of T-cell clonotypes by tracking the read counts of each unique TCRβ sequence across each sorted pool. True antigen-specific clones should be specifically enriched in a unique occupancy pattern that corresponds to the presence of one of the query antigens in K pools. We have reported on methods to map antigens to TCR clonotypes previously (9); we also developed a non-parametric Bayesian model to compute the posterior probability that a given clonotype is statistically associated with a certain antigen in a given experiment. This model uses the available read counts of TCRs to estimate a mean-variance relationship within a given experiment and as well as the probability that a clone will have zero read counts due to incomplete sampling of low frequency clones. This model takes the observed read counts of a clonotype across all N pools and estimates the posterior probability of a clone responding to all possible N choose K addresses and an additional hypothesis that a clone is activated in all pools (truly activated, but not specific to any of our query antigens). To define high-confidence antigen-clone associations, we identified TCR clonotypes assigned to a query antigen from this model with a posterior probability >= 0.9.

## 3 Results

### 3.1 Dataset access

The ImmuneCODE database includes both TCRβ repertoires and MIRA data, and is being shared through the immuneACCESS^®^ data portal at https://clients.adaptivebiotech.com/pub/covid-2020 (DOI 10.21417/ADPT2020COVID). Subjects described in this article can be identified by selecting samples with the “ImmunoCODERelease” tag value “002”.

### 3.2 Repertoire data

The immueRACE study aimed to enroll 1,000 subjects who have been exposed to, are currently infected with, or have recovered from COVID-19. The current release of the database includes T-cell repertoire data from the first 160 participants in the study (including multiple samples from some subjects). This release also includes T-cell repertoire data from 1,326 subjects from global collaborators (**Table 1**).

These data were generated from participant samples using the Adaptive immunosequencing assay (5–7). They include a list of unique TCRβ rearrangements found in each analyzed sample, a count for each rearrangement, TCR sequence features, and sample-level metadata. These samples were not experimentally enriched for SARS-CoV-2-specific T cells, so the majority of TCRβ rearrangements are not involved in the adaptive immune response to this virus. The data can be exported using dedicated links on the immuneACCESS data portal shown above; see **Supplementary Material** for details.

The sequences in each TCR repertoire are annotated with a large set of features that may be useful across different research contexts; **Supplementary Table 1** describes these annotations. In addition, **Supplementary Table 2** provides sample-level metadata, which varies by source and participant but usually includes de-identified subject IDs, COVID-19 status, age in years, and sex. **Supplementary Figure 1** summarizes key demographic features of this table (age, sex, and race) by showing their distributions.

### 3.3 MIRA data

Antigen-associated TCRs were identified using the “Multiplex Identification of Antigen-Specific T-Cell Receptors Assay” (MIRA; 8, 9). MIRA is a high-throughput multiplex assay, enabling the identification of TCRs that bind to large numbers of query antigens (hundreds to thousands at a time and in parallel) by combining immune assays with T-cell receptor sequencing. We use cell sorting based on the upregulation of activation markers to separate a population of antigen-reactive T cells. This positive population is sequenced via the Adaptive TCRβ immunosequencing assay, and clonotypes from activated T cells are identified by enrichment in the positive population compared to a sample of unenriched or unsorted T cells.

With the goal of identifying SARS-CoV-2-specific TCRs, we interrogated T-cell repertoires from both healthy donors and COVID-19 patients. Input cell types varied and included PBMCs from healthy donors or COVID-19 patients, as well as naïve T cells from healthy donors. To maximize TCR yield per experiment, we expanded T cells from both types of input cells for 8-13 days, yielding > 1 billion T cells per donor. When starting with PBMCs from either healthy donors or COVID-19 patients, T cells were expanded polyclonally with soluble anti-CD3. When starting with naïve CD8+ T cells from healthy donors, T cells were expanded following co-culture with monocyte-derived dendritic cells loaded with a pool of all peptides derived from SARS-CoV-2. The point of these expansions is to ensure that every T cell clone is present in every pool of the MIRA experiment. While cell expansion can change the frequencies of different T cell clones relative to the original sample, this does not matter for MIRA as long as clones of interest make it into all of the pools.

We used two different MIRA approaches: peptide- or transgene-based. Both enable the identification of antigen-associated TCRs, however the transgene-based approach enables identification of TCRs that bind epitopes encoded and presented by APCs following expression upon transfection of transgenes. This approach enables us to distinguish the subset of TCRs that respond to endogenously-presented epitopes rather than those that only respond to exogenously loaded peptides; binding or activation following a multimer stain or incubation with peptides may not accurately reflect whether a T cell is specific to an endogenously presented epitope. The underlying assumption for any immunological assay involving multimers or exogenously loaded peptides is that the epitope being tested is actually a presented epitope. For well-characterized epitopes this assumption is reasonable, however when querying large numbers of novel epitopes from a novel virus (SARS-CoV-2, for example) the risk for false positives (defined as TCRs specific to a never-before tested peptide that was exogenously loaded) is higher.

**Figure 1:**
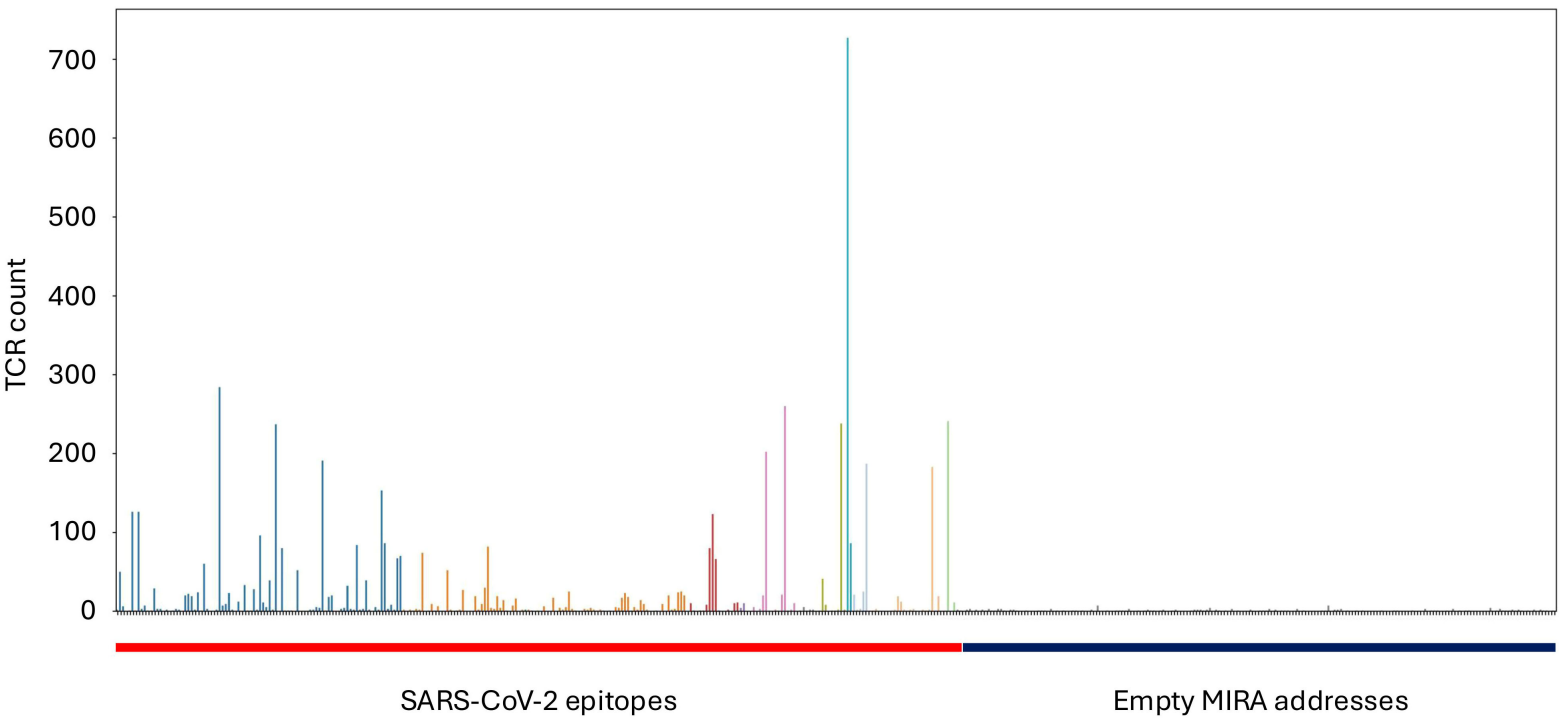
Results from a typical MIRA experiment. Hundreds of “addresses” (subsets of pools) are queried in parallel, and each point on the x-axis corresponds to one address. The height of each bar shows the number of TCR sequences confidently assigned to that address by our statistical model. The addresses on the left side of the figure correspond to SARS-CoV-2 epitopes, while the addresses on the right side were not assigned to any particular antigen.

In total, the MIRA dataset includes more than 160,000 high-confidence SARS-CoV-2-associated TCRs. Results from a typical experiment are shown in **Figure 1**, where each column is a different antigen and the y-axis shows the number of unique TCRs assigned to that antigen. The rightmost 40% of the figure represents MIRA addresses (defined subsets of pools) to which no particular antigen was assigned; these provide a negative control, and can be contrasted with the clear enrichment signal present in several assigned addresses. These TCR-antigen associations are based on statistical modeling of MIRA data; the TCR-antigen pairings have not been verified by separate functional assays, although we expect that most of them are true binders. These MIRA callsets are made available as a set of downloadable files that can be accessed through the immuneACCESS data portal; see **Supplementary Material** for details.

The dataset includes experiments from four MIRA panels. Two of these panels, named “minigene_Set1” and “minigene_Set2”, targeted large protein sequences intended to narrow down which parts of the genome generally elicit an immune response. The other two panels, named “C19_cI” and “C19_cII”, targeted individual peptides or small groups of peptides presented by class I and class II HLA alleles, respectively. Most of the MIRA data included in this release corresponds to the C19_cI panel.

**Supplementary Tables 3** through 10 describe the MIRA data included in the database, as follows: **Supplementary Table 3** (subject-metadata.csv) includes available metadata for each sample from subjects included in the MIRA experiments (including the four minigene and peptide panels described above). HLA types are provided when available. Missing values are generally represented with “N/A”, except for HLA types, where missing data is represented as an empty string. Note that the metadata contained in this file relates to the MIRA results, and is distinct from the TCRβ repertoire metadata referenced in **Supplementary Tables 1** and **2**. **Supplementary Table 4** (orfs.csv) includes the genomic location of the MIRA targets as per GenBank (10). **Supplementary Table 5** (minigene-hits.csv) contains counts of the number of unique TCRs that bound to targets within the “minigene_Set1” and “minigene_Set2” MIRA panels, while **Supplementary Table 6** (minigene- detail.csv) describes the identity of the TCRs bound per target for both minigene MIRA panels. Similarly, **Supplementary Tables 7** and **8** (peptide-hits-ci.csv and peptide-hits-cii.csv) contain counts of the number of unique TCRs that bound to targets within the “C19_cI” and “C19_cII” peptide MIRA panels, while **Supplementary Tables 9** and **10** (peptide-detail-ci.csv and peptide-detail-cii.csv) describe the identity of the TCRs bound per target in these panels. TCRs in **Supplementary Tables 7** and **9** (“ci/CI”) are from CD8+ T cells, whereas TCRs in **Supplementary Tables 8** and **10** (“cii/cII”) are from CD4+ T cells.

## 4 Discussion

To assist in the understanding of the adaptive immune response to SARS-CoV-2, we generated the ImmuneCODE database, which includes a dataset of TCR rearrangements observed in individuals exposed to, infected with, or recovered from COVID-19, and describes the ability of a subset of these TCRs to recognize SARS-CoV-2 epitopes. These data are provided to the scientific community with the goal of contributing to research efforts to develop novel interventions to prevent and treat COVID-19 infections. Though it is not the focus of this paper, the online portal currently includes tools for interacting with the data and integrating new data, although these are not guaranteed to be as stable as the database itself over time, and we expect that most researchers will benefit from downloading the data and building it into their analyses locally.

This resource was first made available to the scientific community in September 2020. Since then, it has been used by researchers around the world to advance our knowledge of SARS-CoV-2 infections and the immunological defenses against them. The ImmuneCODE data have been used to inform vaccine development (11), elucidate immunopathological signatures (12), benchmark tools for predicting TCR-peptide-MHC binding (13), and build diagnostic models of SARS-CoV-2 based on TCR repertoires (14), among many other applications. Any investigator that sequences a TCRβ repertoire can cross-reference it with our MIRA data to identify TCRs that may be responding to SARS-CoV-2 antigens (though a sequence match alone is not sufficient to prove specificity).

The database does have some limitations. All datasets currently available for download are from samples collected prior to September 2020, so they do not reflect any changes in the immune response that may have resulted from subsequent evolution of the virus. In addition, both the immune repertoires and the MIRA data are restricted to TCRβ sequences; the cognate TCRα sequences needed to construct a full T-cell receptor are not included, and a TCRβ sequence alone does not fully determine the antigen specificity of a T-cell clone. The majority of the MIRA data are from CD8+ T cells binding peptides presented by MHC class I molecules. This emphasis provides a rich understanding of which viral epitopes may be most involved in cytotoxic effector responses by CD8+ T cells, but the database is less informative about the targets of CD4+ T cells.

As SARS-CoV-2 continues to circulate and evolve, research into how it affects, and is affected by, the immune system will be ongoing. We anticipate that the ImmuneCODE database will continue to be a valuable resource for these investigations.

## Data Availability

The original contributions presented in the study are publicly available. This data can be found here: https://clients.adaptivebiotech.com/pub/covid-2020.
