# Supplementary material for: A large-scale database of T-cell receptor beta sequences and binding associations from natural and synthetic exposure to SARS-CoV-2
Source: Front Immunol. 2025 Feb 17;16:1488851. doi: 10.3389/fimmu.2025.1488851 (PMC11873104; doi:10.3389/fimmu.2025.1488851)
Supplement: Supplementary file 1 [file DataSheet1.pdf]

Supplementary Material

All data described in this study are available for download from the immuneACCESS data portal at https://clients.adaptivebiotech.com/pub/covid-2020. Supporting files include the following:

- **ImmuneCODE-Repertoires-002.tgz** – A gzipped archive containing 1,414 .tsv files of TCRβ repertoires from subjects exposed to or infected with the SARSCoV-2 virus. The data schema is described in Supplementary Table 1. *Note: This is a large (>20 GB) download.*

- **ImmuneCODE-Repertoires-002.2.tgz** – A gzipped archive containing an additional 74 .tsv files of TCRβ repertoires from subjects exposed to or infected with the SARSCoV-2 virus; two of these files are from samples without metadata. The data schema is described in Supplementary Table 1. *Note: This is a large (~1 GB) download.*

- **ImmuneCODE-Repertoire-Tags-002.2.tsv** – Sample metadata for 1,486 TCRβ repertoires in the gzipped archives shown above. Further details provided in the caption for Supplementary Table 2.

- **ImmuneCODE-MIRA-Release002.1.zip** – Zipped archive containing the following MIRA-related files (further details provided in supplementary table descriptions below):

    - **subject-metadata.csv** – Metadata for subjects included in the MIRA data (Supplementary Table 3)
    - **orfs.csv** – Genomic location of MIRA targets (Supplementary Table 4)
    - **minigene-hits.csv** – Number of TCRs bound per target in the minigene MIRA panels (Supplementary Table 5)
    - **minigene-detail.csv** – Identity of TCRs bound per target in the minigene MIRA panels (Supplementary Table 6)
    - **peptide-hits-ci.csv** – Number of TCRs bound per target in the HLA class I peptide MIRA panel (Supplementary Table 7)
    - **peptide-hits-cii.csv** – Number of TCRs bound per target in the HLA class II peptide MIRA panel (Supplementary Table 8)
    - **peptide-detail-ci.csv** – Identity of TCRs bound per target in the HLA class I peptide MIRA panel (Supplementary Table 9)
    - **peptide-detail-cii.csv** – Identity of TCRs bound per target in the HLA class II peptide MIRA panel (Supplementary Table 10)

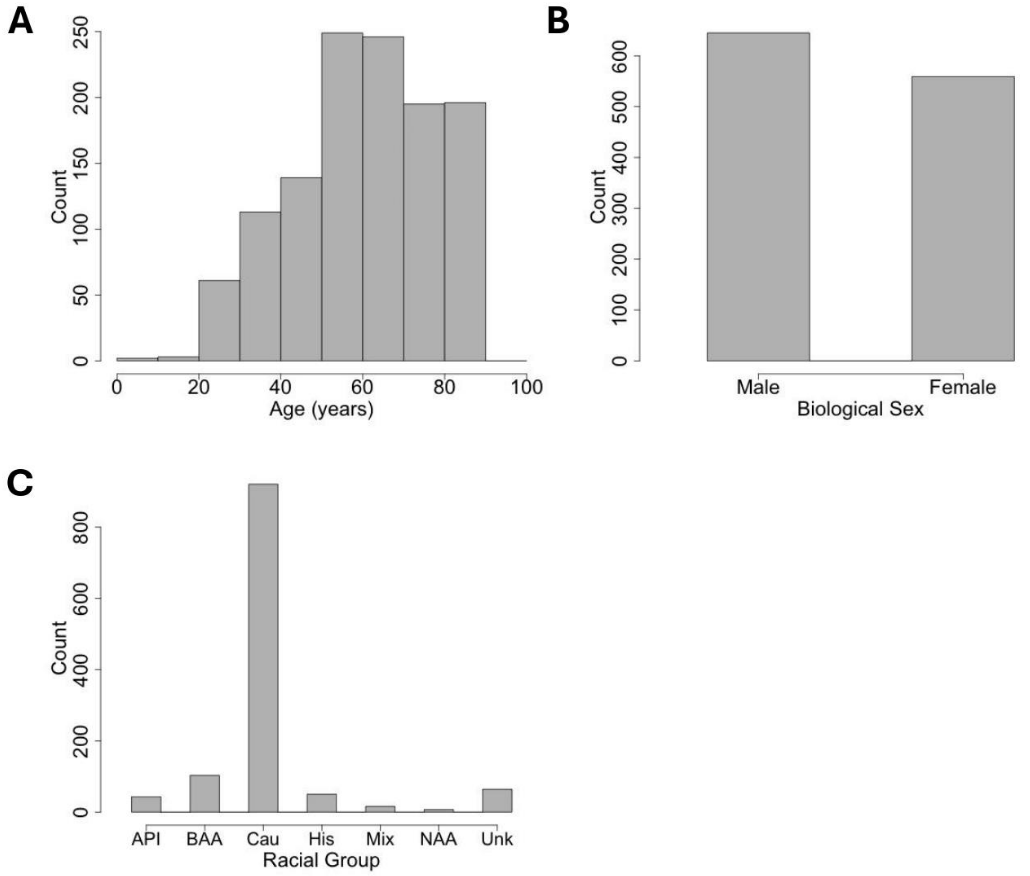

Supplementary Figure 1. Distributions of key demographic parameters of TCRβ repertoire donors. (A) Age of subject, in years. **(B)** Biological sex. **(C)** Racial group; Asian or Pacific Islander (API), Black or African American (BAA), Caucasian (Cau), Hispanic (His), Mixed racial group (Mix), Native American or Alaska Native (NAA), or Unknown racial group (Unk).

Supplementary Table 1. Data schema for TCRβ sequence repertoires. This schema is used in .tsv files (one file per sample/repertoire) in the ImmuneCODE-Repertoires*.tgz download packages.

**Field**	**Data type**	**Description**
rearrangement	string	A particular nucleotide sequence generated through V(D)J recombination, as detected by the Adaptive immunosequencing assay.
extended_rearrangement	string	The full length TCR imputed via algorithm for the rearrangement; includes the full CDR1, CDR2 and CDR3 region.
bio_identity	string	T cell bioidentity refers to the overall protein sequence of a T cell receptor. This is defined as the identified V gene, the amino acid sequence of the CDR3 region and the identified J gene. Two rearrangements with the same bioidentity would be expected to demonstrate the same binding and activation behavior. Data Format: [Amino Acid]+[v_gene]+[j_gene]
amino_acid	string	The amino acid translation of the unique nucleotide rearrangement in the identified CDR3 region. Only productive rearrangements can be translated. Productive rearrangements are in-frame, do not contain a stop codon and can produce a functional protein receptor.
templates	integer	The total number of templates for a specific rearrangement in the sample.
frame_type	enum (In, Out, Stop)	The functional state of a rearranged CDR3: in-frame (productive), out-of-frame, or containing a stop codon.
rearrangement_type	string	The type of rearrangement process that generated a specific rearrangement.

productive_frequency	fraction (0.0-1.0)	The frequency of a specific productive rearrangement among all productive rearrangements within a sample. Calculated as the templates for a specific rearrangement divided by the sum of productive templates for a sample.
cdr1_start_index	integer	The index into the extended_rearrangement string at which the CDR1 region begins.
cdr1_rearrangement_length	integer	The length (in characters) of the CDR1 region within extended_rearrangement.
cdr2_start_index	integer	The index into the extended_rearrangement string at which the CDR2 region begins.
cdr2_rearrangement_length	integer	The length (in characters) of the CDR2 region within extended_rearrangement.
cdr3_start_index	integer	The index into the extended_rearrangement string at which the CDR3 region begins.
cdr3_rearrangement_length	integer	The length of the CDR3 in nucleotides, starting from the first base of the codon for the conserved cysteine in the V gene through the last base of the codon for the conserved residue in the J gene that ends the CDR3.
v_index	integer	The index within the full nucleotide sequence that denotes the Cysteine beginning the CDR3.
n1_index	integer	The index within the full nucleotide sequence that denotes the start of the N1 (VD) region.
d_index	integer	The index within the full nucleotide sequence that denotes the start of the D region.

n2_index	integer	The index within the full nucleotide sequence that denotes the start of the N2 (DJ) region.
j_index	integer	The index within the full nucleotide sequence that denotes the start of the J region.
v_deletions	integer	The number of nucleotides deleted from the V gene during recombination.
n1_insertions	integer	The number of nucleotides inserted in the N1 (VD) junction during recombination.
d3_deletions	integer	The number of nucleotides deleted from the 3' end of the D gene during recombination.
d5_deletions	integer	The number of nucleotides deleted from the 5' end of the D gene during recombination.
n2_insertions	integer	The number of nucleotides inserted in the N2 (DJ) junction during recombination.
j_deletions	integer	The number of nucleotides deleted from the J gene during recombination.
chosen_j_allele	string	The j-gene allele that was used to impute extended_rearrangement.
chosen_j_family	string	The j-gene family that was used to impute extended_rearrangement.
chosen_j_gene	string	The j-gene that was used to impute extended_rearrangement.
chosen_v_allele	string	The v-gene allele that was used to impute extended_rearrangement.

chosen_v_family	string	The v-gene family that was used to impute extended_rearrangement.
chosen_v_gene	string	The v-gene that was used to impute extended_rearrangement.
d_allele	string	The identified D gene allele that contributed to a specific rearrangement.
d_allele_ties	csv	A comma-separated list of equivalently-scored D gene alleles identified during annotation.
d_family	string	The identified D gene family that contributed to a specific rearrangement.
d_family_ties	csv	A comma-separated list of equivalently-scored D gene families identified during annotation.
d_gene	string	The identified D gene that contributed to a specific rearrangement.
d_gene_ties	csv	A comma-separated list of equivalently-scored D genes identified during annotation.
d_resolved	string	A concise string identifying the most specific D gene family, gene or allele identified during annotation.
j_allele	string	The identified J gene allele that contributed to a specific rearrangement.
j_allele_ties	csv	A comma-separated list of equivalently-scored J gene alleles identified during annotation.

j_family	string	The identified J gene family that contributed to a specific rearrangement.
j_family_ties	csv	A comma-separated list of equivalently-scored J gene families identified during annotation.
j_gene	string	The identified J gene that contributed to a specific rearrangement.
j_gene_ties	csv	A comma-separated list of equivalently-scored J genes identified during annotation.
j_resolved	string	A concise string identifying the most specific J gene family, gene or allele identified during annotation.
v_allele	string	The identified V gene allele that contributed to a specific rearrangement.
v_allele_ties	csv	A comma-separated list of equivalently-scored V gene alleles identified during annotation.
v_family	string	The identified V gene family that contributed to a specific rearrangement.
v_family_ties	csv	A comma-separated list of equivalently-scored V gene families identified during annotation.
v_gene	string	The identified V gene that contributed to a specific rearrangement.
v_gene_ties	csv	A comma-separated list of equivalently-scored V genes identified during annotation.

v_resolved	string	A concise string identifying the most specific V gene family, gene or allele identified during annotation.

Supplementary Table 2. Sample metadata for 1,486 TCRβ repertoires. This table includes all available metadata for samples with .tsv files in the ImmuneCODE-Repertoires*.tgz download packages. Metadata rows can be mapped to repertoire .tsv files via the *sample_name* column; each sample ID is included in the name of the corresponding repertoire .tsv file.

This supplementary table is available for download from the ImmuneACCESS data portal at https://clients.adaptivebiotech.com/pub/covid-2020; it is the file named **ImmuneCODE-Repertoire-Tags-002.2.tsv**.

Supplementary Table 3. Metadata for subjects included in the MIRA data. The schema below describes the elements of a supplementary table that can be downloaded from the ImmuneACCESS data portal at https://clients.adaptivebiotech.com/pub/covid-2020; it is the file named **subject-metadata.csv** within the **ImmuneCODE-MIRA-Release002.1.zip** download package.

**Field**	**Data type**	**Description**
Experiment	string	Opaque identifier for the MIRA experiment. This column joins to the *-detail*.csv files.
Subject	string	Opaque identifier for the subject (also the “sample” in the context of MIRA).
Cell Type	enum	· PBMC<br>· naive_CD8
Target Type	enum	The MIRA panel for the experiment:<br>· minigene_Set1<br>· minigene_Set2<br>· C19_cI (class I-presented peptides)<br>· C19_cII (class II-presented peptides)
Cohort	enum	· Healthy (No known exposure)<br>· COVID-19-Convalescent<br>· COVID-19-Acute<br>· COVID-19-Exposed
Age	integer	In years or N/A
Gender	enum	· M<br>· F<br>· N/A (other)
Race	string	Uncontrolled values or N/A
HLA	multiple columns	HLA values as provided by the data source.

Supplementary Table 4. Genomic location of MIRA targets. The schema below describes the elements of a supplementary table that can be downloaded from the ImmuneACCESS data portal at https://clients.adaptivebiotech.com/pub/covid-2020; it is the file named **orfs.csv** within the **ImmuneCODE-MIRA-Release002.1.zip** download package.

**Field**	**Data type**	**Description**
orf	string	The abbreviated name of an open reading frame. Joins to the "ORF"-related columns in *-hits*.csv and *-detail*.csv files.
index_genome	integer	The 1-based index of the first base of the ORF within the genome.
end_index_inclusive	integer	The 1-based index of the last base of the ORF within the genome.

Supplementary Table 5. Number of TCRs bound per target in the minigene MIRA panels. The schema below describes the elements of a supplementary table that can be downloaded from the ImmuneACCESS data portal at https://clients.adaptivebiotech.com/pub/covid-2020; it is the file named **minigene-hits.csv** within the **ImmuneCODE-MIRA-Release002.1.zip** download package.

**Field**	**Data type**	**Description**
ORF	string	The ORF in which this target is located.
ORF Genebank ID	string	The identifier for the sequence from which the target was selected.
Amino Acid	string	The protein sequence of the target.
Start Index in Genome	integer	The 1-based index of the first base of the target within the genome.
End Index in Genome	integer	The 1-based index of the last base of the target within the genome.
Hits	integer	The unique count of TCRs identified as binding to the target, across all experiments.

Supplementary Table 6. Identity of TCRs bound per target in the minigene MIRA panels. The schema below describes the elements of a supplementary table that can be downloaded from the ImmuneACCESS data portal at https://clients.adaptivebiotech.com/pub/covid-2020; it is the file named **minigene-detail.csv** within the **ImmuneCODE-MIRA-Release002.1.zip** download package.

**Field**	**Data type**	**Description**
TCR BioIdentity	string	Represents the overall protein sequence of a T cell receptor. This is defined as the identified V gene, the amino acid sequence of the CDR3 region and the identified J gene. Two rearrangements with the same bioidentity would be expected to demonstrate the same binding and activation behavior. Data format: [Amino Acid]+[v_gene]+[j_gene]
TCR Nucleotide Sequence	string	The unique TCRB sequence identified as binding to the target.
Experiment	string	The experiment in which the binding was observed (joins to the subject-metadata.csv file).
ORF	string	The ORF in which this minigene target is located.
ORF Genebank ID	string	The identifier for the sequence from which the target was selected.
Amino Acid	string	The protein sequence of the minigene target.
Start Index in Genome	integer	The 1-based index of the first base of the target within the genome.
End Index in Genome	integer	The 1-based index of the last base of the target within the genome.

Supplementary Table 7. Number of TCRs bound per target in the HLA class I peptide MIRA panel. The schema below describes the elements of a supplementary table that can be downloaded from the ImmuneACCESS data portal at https://clients.adaptivebiotech.com/pub/covid-2020; it is the file named **peptide-hits-ci.csv** within the **ImmuneCODE-MIRA-Release002.1.zip** download package.

Supplementary Table 8. Number of TCRs bound per target in the HLA class II peptide MIRA panel. The schema below describes the elements of a supplementary table that can be downloaded from the ImmuneACCESS data portal at https://clients.adaptivebiotech.com/pub/covid-2020; it is the file named **peptide-hits-cii.csv** within the **ImmuneCODE-MIRA-Release002.1.zip** download package.

**Field**	**Data type**	**Description**
ORF	string	The ORF in which this target is located.
Amino Acids	string	The protein sequence of the target.
Start Index in Genome	integer	The 1-based index of the first base of the target within the genome.
End Index in Genome	integer	The 1-based index of the last base of the target within the genome.
Hits	integer	The unique count of TCRs identified as binding to the target, across all experiments.

Supplementary Table 9. Identity of TCRs bound per target in the HLA class I peptide MIRA panel. The schema below describes the elements of a supplementary table that can be downloaded from the ImmuneACCESS data portal at https://clients.adaptivebiotech.com/pub/covid-2020; it is the file named **peptide-detail-ci.csv** within the **ImmuneCODE-MIRA-Release002.1.zip** download package.

Supplementary Table 10. Identity of TCRs bound per target in the HLA class II peptide MIRA panel. The schema below describes the elements of a supplementary table that can be downloaded from the ImmuneACCESS data portal at https://clients.adaptivebiotech.com/pub/covid-2020; it is the file named **peptide-detail-cii.csv** within the **ImmuneCODE-MIRA-Release002.1.zip** download package.

**Field**	**Data type**	**Description**
TCR BioIdentity	string	Represents the overall protein sequence of a T cell receptor. This is defined as the identified V gene, the amino acid sequence of the CDR3 region and the identified J gene. Two rearrangements with the same bioidentity would be expected to demonstrate the same binding and activation behavior. Data format: [Amino Acid]+[v_gene]+[j_gene]
TCR Nucleotide Sequence	string	The unique TCRB sequence identified as binding to the target.
Experiment	string	The experiment in which the binding was observed (joins to the subject-metadata.csv file).
ORF Coverage	string	The ORF(s) in which this target is located.
Amino Acids	string	The protein sequence of the target.
Start Index in Genome	integer	The 1-based index of the first base of the target within the genome.
End Index in Genome	integer	The 1-based index of the last base of the target within the genome.
